# Fluorinated Silane-Modified Filtroporation Devices Enable Gene Knockout in Human Hematopoietic Stem and Progenitor Cells

**DOI:** 10.1021/acsami.3c07045

**Published:** 2023-08-24

**Authors:** Isaura M. Frost, Alexandra M. Mendoza, Tzu-Ting Chiou, Philseok Kim, Joanna Aizenberg, Donald B. Kohn, Satiro N. De Oliveira, Paul S. Weiss, Steven J. Jonas

**Affiliations:** † Department of Bioengineering, University of California, Los Angeles, Los Angeles, California 90095, United States; ‡ UCLA Medical Scientist Training Program, David Geffen School of Medicine, University of California, Los Angeles, Los Angeles, California 90095, United States; § Department of Pediatrics, David Geffen School of Medicine, University of California, Los Angeles, Los Angeles, California 90095, United States; ∥ Department of Chemistry and Biochemistry, University of California, Los Angeles, Los Angeles, California 90095, United States; ⊥ California NanoSystems Institute, University of California, Los Angeles, Los Angeles, California 90095, United States; # John A. Paulson School of Engineering and Applied Sciences, Harvard University, Cambridge, Massachusetts 02138, United States; ¶ Department of Molecular and Medical Pharmacology, University of California, Los Angeles, Los Angeles, California 90095, United States; ∇ Department of Microbiology, Immunology and Molecular Genetics, University of California, Los Angeles, Los Angeles, California 90095, United States;; ○ Eli and Edythe Broad Center of Regenerative Medicine and Stem Cell Research, University of California, Los Angeles, Los Angeles, California 90095, United States;; ◆ Department of Materials Science and Engineering, University of California, Los Angeles, Los Angeles, California 90095, United States;; $ Children’s Discovery and Innovation Institute, University of California, Los Angeles, Los Angeles, California 90095, United States

**Keywords:** filtroporation, intracellular delivery, hematopoietic stem cells, gene knockout, gene therapy

## Abstract

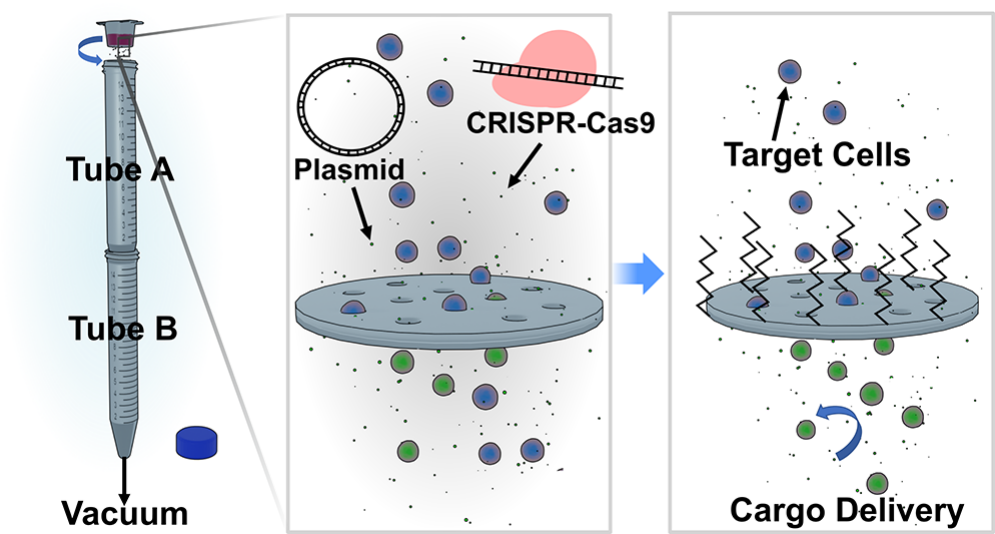

Intracellular delivery technologies that are cost-effective, non-cytotoxic, efficient, and cargo-agnostic are needed to enable the manufacturing of cell-based therapies as well as gene manipulation for research applications. Current technologies capable of delivering large cargoes, such as plasmids and CRISPR-Cas9 ribonucleoproteins (RNPs), are plagued with high costs and/or cytotoxicity and often require substantial specialized equipment and reagents, which may not be available in resource-limited settings. Here, we report an intracellular delivery technology that can be assembled from materials available in most research laboratories, thus democratizing access to intracellular delivery for researchers and clinicians in low-resource areas of the world. These filtroporation devices permeabilize cells by pulling them through the pores of a cell culture insert by the application of vacuum available in biosafety cabinets. In a format that costs less than $10 in materials per experiment, we demonstrate the delivery of fluorescently labeled dextran, expression plasmids, and RNPs for gene knockout to Jurkat cells and human CD34^+^ hematopoietic stem and progenitor cell populations with delivery efficiencies of up to 40% for RNP knockout and viabilities of >80%. We show that functionalizing the surfaces of the filters with fluorinated silane moieties further enhances the delivery efficiency. These devices are capable of processing 500,000 to 4 million cells per experiment, and when combined with a 3D-printed vacuum application chamber, this throughput can be straightforwardly increased 6–12-fold in parallel experiments.

## Introduction

Methodologies that enable efficient, cost-effective, and non-cytotoxic intracellular delivery of clinically relevant biomolecules are paving the way for exciting medical interventions that leverage advances in genome editing and engineering. These cell-based treatments, such as gene-modified stem cell and chimeric antigen receptor (CAR) T-cell strategies, are increasingly offering therapeutic solutions to genetic diseases and cancers, respectively.^1−3^ In particular, allogeneic hematopoietic stem-cell transplantation has been the single curative option for those suffering from monogenic blood disorders, such as sickle cell disease, β-thalassemia, and primary immunodeficiencies, but issues with donor matching and graft-versus-host disease limit this approach.^4^ Autologous gene therapies are elegant and promising alternatives, whereby the patient’s own cells are modified at the genomic level to correct genotypes and alleviate disease phenotypes. To date, most clinical progress has been made in the field of viral vector-mediated gene modification,^1,2^ which harnesses viruses’ natural ability to enter cells and to modify DNA. The manufacturing of these viral-based therapies has been burdened with extremely high costs, while populations that are frequently affected by prevalent hematological disorders are often located in medically underserved and/or low-resource regions of the world,^5^ underscoring the need for intracellular delivery technologies that are accessible and easy to use and require little training to operate.^6,7^ Additionally, issues with potential insertional mutagenesis due to semirandom gene insertion mediated by viral carriers have driven the gene-editing field away from utilizing viral vectors and toward more targeted strategies such as those employing zinc-finger nucleases, transcription activator-like effector nucleases (TALENs), clustered regularly interspaced palindromic repeats (CRISPR)-Cas, and, more recently, prime and base editors.^1,8,9^ However, these important gene-modifying biomolecules are often large proteins that need to be delivered to cells using non-cytotoxic and effective intracellular delivery strategies, because the latest favored viral vectors suffer from size limitations and are thus unable to carry the large DNA constructs encoding these proteins.^1,10−12^ Additionally, gene manipulation through targeted knockouts is an important research tool to elucidate functional gene roles and pathways that may inform clinical targets and outcomes.^13^

Commercially available techniques such as lipofection and electroporation (or nucleofection) are well established but can be cytotoxic and difficult to scale and require expensive specialized equipment and/or reagents.^14−16^ A new favorable biophysical methodology for intracellular delivery was developed over the past decade by Jensen, Langer, and colleagues, our group, and others and it consists of squeezing cells through narrow constrictions 30–80% of their diameter, which has been shown to permeabilize cells transiently, rendering them susceptible to cargo uptake.^17−23^ The mechanism behind this transient cell permeability is not fully understood but presumably relies on a combination of forced repulsive interactions between polar phospholipid head groups because membrane lipids are pushed against one another when they are sheared against the walls of microfluidic devices, thus facilitating the formation of membrane discontinuities, as well as cytosolic egress due to compression of the cell’s volume.^20^ Various cargoes have been delivered in this manner, from small molecules, drugs, fluorescently labeled sugars, and Cas9 ribonucleoproteins (RNPs) to large plasmids and antibodies.^20^ Importantly, this technique has been shown to circumvent issues related to transcriptional abnormalities seen in primary cells treated by electroporation,^22^ potentially offering a healthier alternative to porating cells. One of the limitations of this approach is clogging with cell debris because some cells are destroyed at the inlets of constrictions, variable delivery efficiencies, and the requirement for specialized equipment such as silicon-based microfluidic chips and bulky pressurized gas tanks to drive flow.^17^ Since its emergence, this particular methodology has been broadly researched in laboratories, with creative solutions to limitations from our group and others, including the development of antifouling coatings on poly(dimethylsiloxane)-based devices,^18^ the introduction of pillars to disperse cell populations as they squeeze through obstacles,^24^ fishbone geometries for cell deformation,^25^ repeated cell compression,^26^ and even combining squeezing with electroporation to boost the delivery efficiency and quickly draw cargoes to the nucleus.^23^ Other methodologies include packaging cargoes in nanoparticles,^27−30^ acoustofluidic sonoporation,^19^ nanochannel electroporation,^31,32^ mechanoporation by perforating the cell membrane,^33−37^ and various microfluidics-based approaches.^38−40^ All of these techniques share one limitation, which is the requirement for specialized instrumentation, including facilities to manufacture microfluidic devices that can be costly and require extensive training. The term filtroporation (FP) was coined by Williams et al., who showed that high-molecular-weight dextran and plasmids could be delivered to Chinese hamster ovary cells by forcing them through a porous filter using positive pressure.^41^ Others have sought to utilize similar commercially available porous membranes to devise intracellular delivery devices and have succeeded in demonstrating their role in facilitating cargo delivery.^32,42^ Cao et al. localized electroporation to immortalized cells by making use of commercial nanoporous membranes, thus achieving high delivery efficiencies while preserving the cell viability.^32^ Yen and colleagues also used commercially available filters with micrometer-scale pores to deform human hematopoietic stem and progenitor cells (HSPCs) as they are pushed through the membrane’s holes. This method achieved consistent gene knockout in stem cells while maintaining their differentiation and engraftment potential but utilized large amounts of RNPs (1227 pmol or nearly 25 μM) to achieve editing, crippling the cost-effectiveness of this approach.^42^ Although these two reports utilize readily available membranes, they, nonetheless, require other specialized equipment, such as electrodes for electroporation and bulky custom-made metal holders attached to a pressure gauge and gas system to mount filters and apply pressure to cells. Bearing in mind that populations frequently affected by hemoglobinopathies are often located in low-resource regions of the world,^5^ there remains an unmet need for democratized delivery technologies to enable research and clinical programs in underserved settings.^6,7^

Here, we report a FP approach that can be constructed solely with materials available in most research laboratories. Our FP devices utilize commercially available poly(ethylene terephthalate) (PET) porous filters, available from the manufacturer and mounted on cell culture inserts, as the platform for cell deformation and require only standard conical tubes for cell collection. In contrast to previous embodiments of FP techniques, our method does *not* utilize positive pressure to force cells through the pores but rather a vacuum source, commonly available in biosafety cabinets. We demonstrate the delivery of fluorescently labeled dextran, plasmids, and Cas9 RNPs in immortalized cells and peripheral blood CD34^+^ HSPCs with robust efficiencies while maintaining cellular viability and function. Functional knockout of the CD55 gene is achieved in both cell types but with RNP concentrations that are 1 order of magnitude lower than previously reported for FP strategies^42^ and mainly employs readily available materials for transfection. We explored the roles of hydrophobic surface chemistries by applying fluorinated silane coatings alone or in combination with fluorinated oils to create slippery liquid-infused porous surface (SLIPS) coatings. These SLIPS coatings are a novel class of omniphobic materials created when a porous substrate or polymer brush is combined with a surface-energy-matched lubricant such that the substrate–lubricant system components preferentially interact with one another while repelling any immiscible solution put in contact with the material.^43,44^ We find that the functionalization of filter surfaces with fluorinated silane moieties is sufficient to improve the delivery efficiency of fluorescent dextran and RNPs. We compare our approach to electroporation and report that FP offers a higher knockout efficiency per unit of cargo for both immortalized Jurkat cell lines and primary HSPCs. Importantly, these devices are economical (<$10) and easy to assemble and to operate, requiring little training or specialized equipment. Altogether, these data indicate that this cell deformation platform is a promising strategy that can be applied in laboratories around the world to effect efficient gene editing in hard-to-transfect cell types applied in research and in the generation of emerging gene therapies. When operating multiple FP devices in parallel, we are currently able to process 3–6 million cells within seconds (500,000 cells per condition). Given its modularity and customizable nature enabled by 3D-printing strategies, we envision that massive parallelization of this approach can be readily accomplished to suit the needs of researchers. Here, we demonstrate, for the first time, to our knowledge, a mechanical deformation-based intracellular delivery method that employs a negative pressure gradient across a membrane to permeabilize target cells.

## Results and Discussion

### Device Manufacturing and Setup

Filtroporation devices were designed to deform the plasma membrane as cells are forced through well-defined pores smaller than their own diameter. Based on evidence by our group and others that cell membrane compression can result in transient permeability enabling biomolecular cargo delivery,^17,18,24,42,45^ we devised a device that employs commercially available PET track-etched filters mounted on cell culture inserts as the structure inducing cell deformation. The filters used had pores of 8 μm in diameter with thicknesses of ca. 7 μm. Cells with diameters of ca. 11 μm were chosen as target cells to enable sufficient cell deformation for intracellular delivery while preventing fatal cell shearing or bursting. To propel cells through the pores, negative pressure was used, given the ease of access to a source of vacuum in laboratory biosafety cabinets and elsewhere.

**Figure 1:**
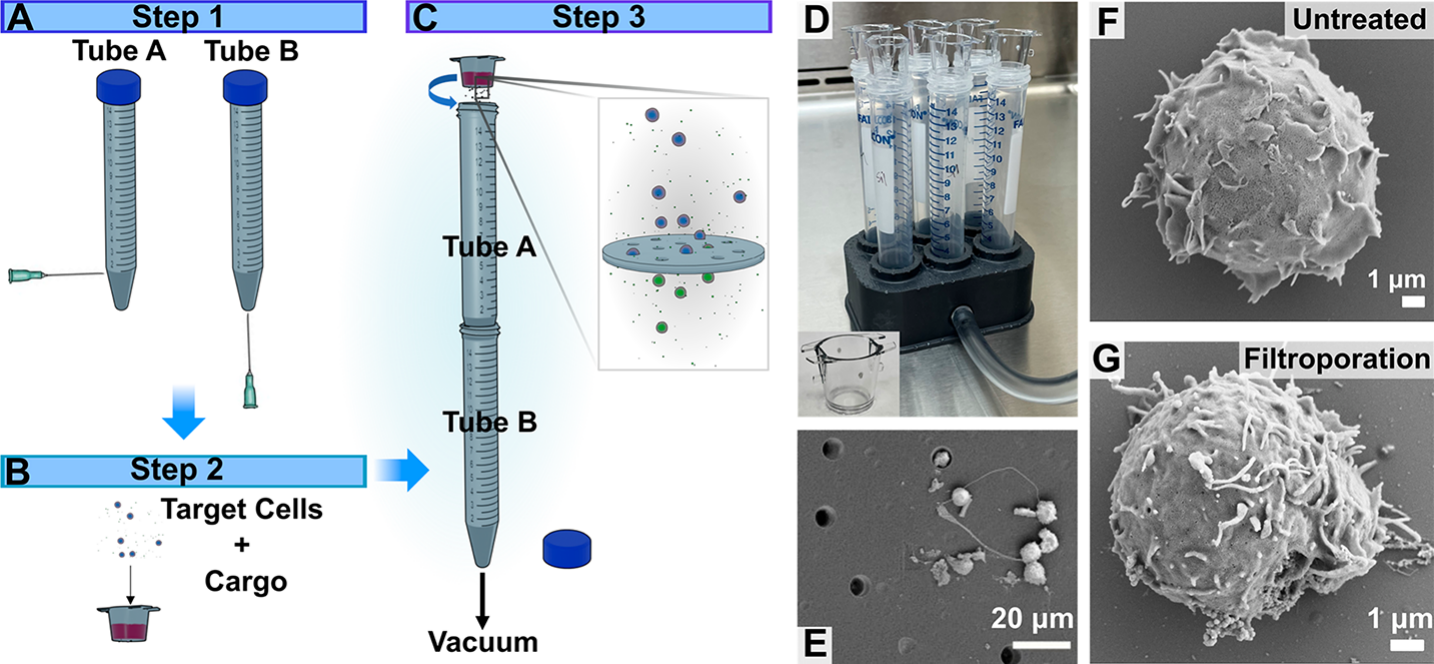
Schematic of the filtroporation (FP) device assembly and parallelized system and scanning electron microscope (SEM) images of filters and cells. (A) First, two conical tubes are perforated with needles. (B) A commercially available 12-well cell culture insert containing a poly(ethylene terephthalate) (PET) track-etched membrane with 8 μm pores is then loaded with target cells and biomolecular cargo in an appropriate buffer. (C) The device is assembled by placing tube A through the opening of tube B to form an airtight seal, and the loaded insert is placed to the opening of tube A to form an airtight seal; a house vacuum is applied to the perforation of tube B and pulls the cell mixture through the pores of the inset’s membrane. (D) A 3D-printed chamber is developed to enable the application of vacuum to six inserts at a time; the chamber connects to the vacuum via its side port. The inset shows a photograph of the inserts available from the manufacturer. (E) Scanning electron microscope images of porous membranes and cells passing through the pores; cells were fixed immediately following FP treatment. Cell morphology (F) before and (G) after treatment, visualized using SEM.

The setup to collect the cells as they pass through the pores can also be assembled from materials routinely found in most laboratories, namely, two 15 mL conical tubes that are assembled in pairs. To enable vacuum to be applied to the insert containing the cell suspension, a perforation was introduced in tube A by a 20-gauge sterile needle near the 1 mL mark and another perforation placed on the flat bottom of tube B (Figure 1A). The perforation in tube A is positioned such that it fits within the opening of tube B after insertion; we found that puncturing of the tube just below (1 mm) the 1 mL mark was appropriate given that the height of the puncture limits the maximum volume of the cell suspension that can be used in experiments, and lower placements can result in a spillover of the cell mixture through the hole. Next, the cell culture insert is loaded with the cell suspension containing the target cells mixed with the desired delivery cargo in an appropriate buffer [e.g., a fetal bovine serum (FBS)-free cell culture medium; Figure 1B]. Given that some solution volume is lost as cells are pulled through the filters due to splashing on the internal surfaces of the collection tube, a total volume of 200 μL was found to be optimal for experiments because it maximizes cell recovery and minimizes the amount of cargo required to achieve the desired final cargo concentration. For the setup described herein, 12-well cell culture inserts were best suited because they fit within the opening of 15 mL conical tubes, forming airtight seals. The loaded insert can then be placed in the opening of tube A and a flexible piece of tubing connected to the opening of tube B and the vacuum source of the biosafety cabinet; the application of negative pressure (−20″ Hg) drives the cell suspension through the track-etched pores, and the cell suspension is collected within tube A (Figure 1C). Given the reliance of our system on the vacuum available in biosafety cabinets, we only tested vacuum pressures ranging to the maximum pressure of −20″ Hg by utilization of a pressure release valve. Our studies with T cells showed no differences in cytotoxicity or delivery efficiency with decreased pressure (Figure S1). Due to high background (for “Incub Ctrl” conditions) and low delivery efficiency in T cells, no further experiments were performed with this cell type. To achieve higher throughput and enable multiple conditions to be run at a time, we designed and 3D-printed a chamber using a stereolithographic 3D printer (FormLabs, flexible resin) containing 6 or 12 slots for conical tubes, permitting the application of vacuum to all tubes simultaneously (Figures 1D and S2A). Importantly, these devices are economical (<$10 per experiment), can be promptly and easily assembled by most research laboratories, and are free of the need for costly specialized instrumentation.

### Membrane and Cell Surface Characterization by Electron Microscopy

Filter membranes were imaged with scanning electron microscopy (SEM) to measure the pore size and to observe the pore distribution and membrane thickness. The filters were found to have random pore distributions and consistent pore diameters of 8 μm, as reported by the manufacturer (Figures 1E and S2B). To study the membrane and cells after the application of vacuum, Jurkat cells were filtroporated and inserts immediately fixed and prepared for SEM imaging. Cells can be observed going through the pores or remaining on the top surface of the filters between pores (Figure 1E). To examine the cell morphology and to probe whether FP results in superficial cell damage, Jurkat cells were again passed through the pores of a device and collected in a tube containing a glutaraldehyde fixative solution. Cells were subsequently glued onto a piece of silicon oxide, and samples were prepared for SEM by sequential dehydration and critical point drying. At least five cells were imaged for each condition per duplicate experiments, and SEM images showed no significant differences between the treated and untreated samples (Figure 1F,G), suggesting that FP does not cause morphological damage to the cells.

### Intracellular Delivery by Filtroporation in Model Cells

**Figure 2:**
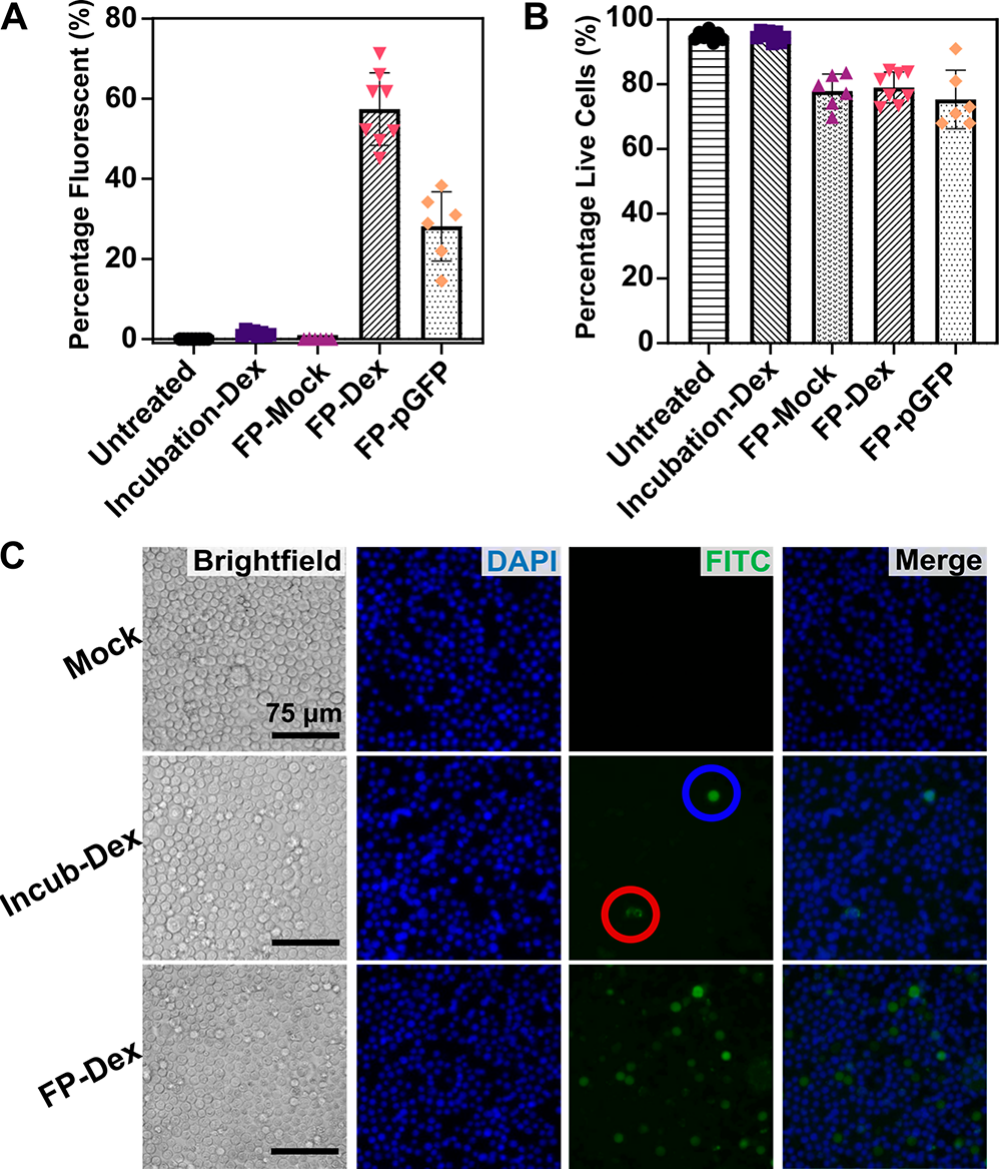
FP enabling the delivery of fluorescent cargo and plasmids to Jurkat cells. (A) Delivery efficiency of FITC-tagged dextran (FP-Dex, 40 kDa) and eGFP-encoding plasmids (FP-pGFP). Controls were either untreated, incubated with dextran (Incubation-Dex), or filtroporated without cargo (FP-Mock). (B) Cell viabilities at 72 h after transfection as determined by trypan blue (TB) counterstaining or DAPI. *N* = 4 independent FITC-Dex experiments and *N* = 6 independent pGFP experiments. (C) Fluorescence microscopy of cells 2 h after treatment by FP without cargo (Mock), incubation with FITC-dextran (Incub-Dex), or FP with FITC-dextran (FP-Dex). Dye internalization can be seen as brightly fluorescent cells (blue circle), while adhesion of FITC-Dex to the outer cell membrane can be seen as discontinuous fluorescent dots (red circle).

To test whether this system could be used to permeabilize cells transiently and to enable biomolecular cargo delivery, we first sought to deliver fluorescently tagged dextran to model cells. Jurkat cells (500,000 to 4 million) were resuspended in 200 μL of a FBS-free Roswell Park Memorial Institute (RPMI) medium containing 300 μg/mL fluorescein isothiocyanate (FITC)-dextran (FITC-Dex) with a molecular weight of 40 kDa. Transfection in serum was tested, and we observed that the application of vacuum in serum conditions causes the generation of foam in the cell suspension, resulting in increased cell loss. Thus, all subsequent experiments were performed in serum-free conditions. Filtroporated cells were allowed to recover for 15 min to provide enough time for the cell membrane to reconstitute before subsequent characterization experiments were performed. After recovery, cells were washed with 1× phosphate-buffered saline (PBS), and their fluorescence was measured by flow cytometry shortly thereafter (<2 h). After dead cell exclusion by 4′,6-diamidino-2-phenylindole (DAPI) staining, 57 ± 9% of cells were FITC positive, with an average viability of 79 ± 5% (FP-Dex). Mock treatment (FP-Mock) and incubation with fluorescent molecules (Incubation-Dex) yield little background fluorescence (0.042 ± 0.088% and 1.4 ± 0.6%, respectively; Figures 2A and S3A) and viabilities of 78 ± 5% and 95 ± 2% (Figure 2B), respectively. Fluorescence microscopy imaging was performed to test that the FITC signal can be attributed to internalization of fluorescent dextran and is not simply caused by adhesion of the molecules to the extracellular side of the cell membranes (Figure 2C) immediately following flow cytometry. Incubation with dextran (Incub-Dex) showed some cells with bright FITC fluorescence (blue circle), likely indicating internalization of FITC cargo by endocytosis, and also some clusters of fluorescence (red circle) attributed to the attachment of fluorescent molecules to cell surfaces. Filtroporated cells (FP-Dex) are observed to display uniform fluorescence only, suggesting internalization of cargo. Given the short turnaround time between flow cytometry and imaging, DAPI staining of live cells may not have been complete by the time those images were acquired and thus marks dead cells in this experiment. Taken together, these data indicate a successful intracellular delivery by FP with robust cell viability, even after short recovery times.

Next, we investigated whether our system was suited to deliver more complex biomolecular cargos not only to the cytoplasm but also to the cellular nucleus. To test this capability, we prepared Jurkat suspensions (4 million cells) in FBS-free RPMI containing 0.1 mg/mL cytomegalovirus (CMV)-driven enhanced green fluorescent protein (eGFP)-encoding plasmids. This concentration was chosen to match other reported cell squeezing platforms.^17,19^ After FP, cells were cultured in a complete medium for up to 72 h, with the cell density and viability estimated daily by trypan blue (TB) counterstaining. Expression of GFP was visualized by confocal microscopy (Figure S4) and determined by flow cytometry, peaking at 72 h and averaging 28 ± 9% across experiments (FP-pGFP; Figures 2A and S3B), while the viabilities of treated cells remained >75% at that time point (Figure 2B). Droplet digital polymerase chain reaction (ddPCR) of reverse-transcribed mRNA extracts showed that GFP mRNA was present in treated cell populations but not mock samples, providing further support of the successful delivery and expression of the plasmids (Figure S2C). We observed expression of GFP by fluorescence microscopy as early as 4 h after FP; given that plasmids need to translocate to the nucleus for GFP protein expression, these data suggest that FP may cause nuclear membrane permeabilization in addition to plasma membrane permeabilization. We also observed this behavior during acoustofluidic sonoporation of Jurkat cells and HSPCs.^19^

### Chemical Modification of Filters

**Figure 3:**
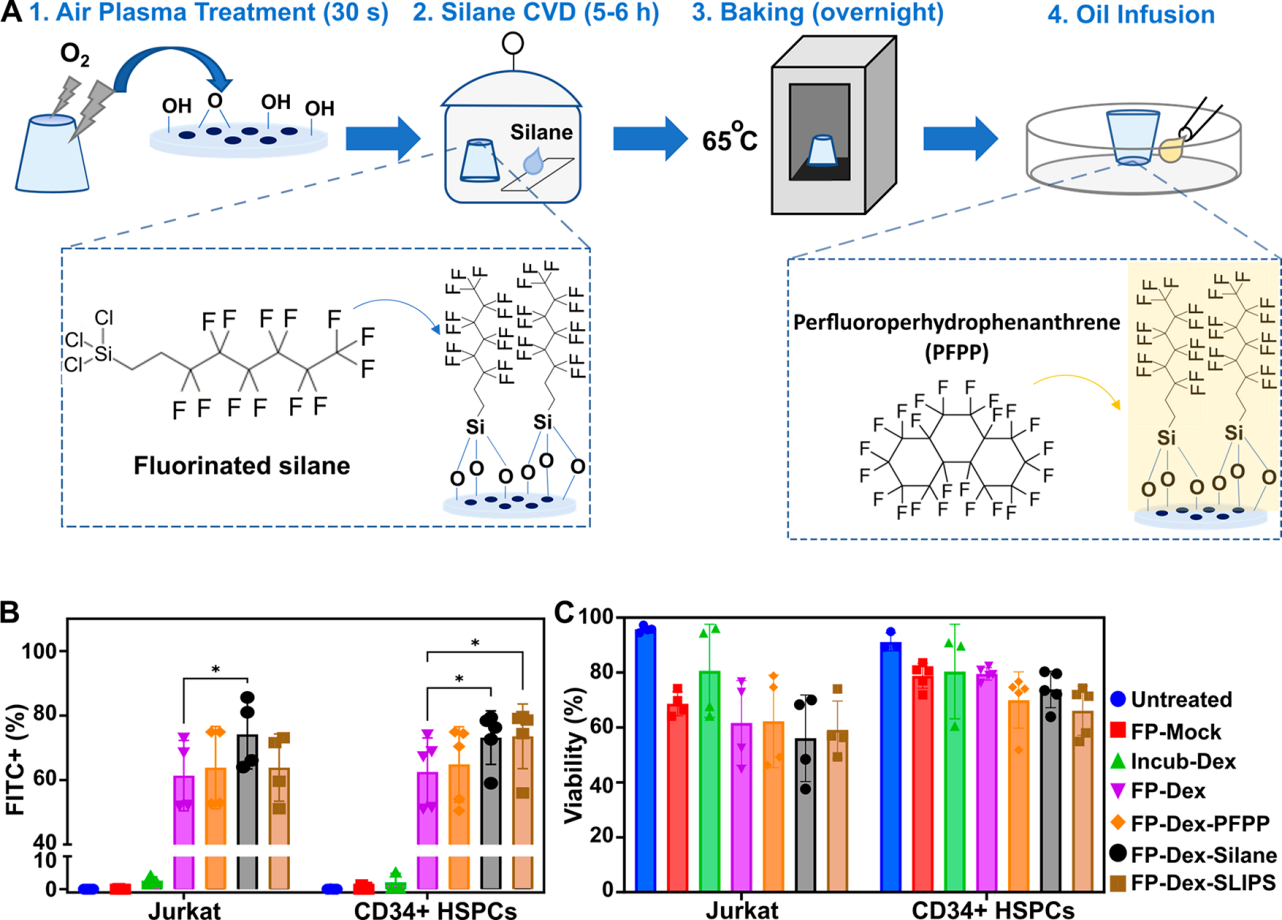
Treatment with silane improving the delivery of cargo to Jurkat cells and human hemopoietic stem and progenitor cells (HSPCs). (A) Schematic representation of surface treatment with TPFS followed by PFPP infusion to create SLIPS. (B) Delivery efficiency of 40 kDa FITC-Dex by FP as determined by flow cytometry within 1 h of treatment. Controls were either untreated, filtroporated without cargo (FP-Mock), or incubated with FITC-Dex (Incub-Dex). Inserts were either untreated (FP-Dex), treated with PFPP only without silanization (FP-Dex-PFPP), and silanized only without oil (FP-Dex-Silane) or both silane and oil (FP-Dex-SLIPS). (C) Cell viabilities after transfection as determined by DAPI staining during flow cytometry. *N* = 4 independent experiments with Jurkat cells and *N* = 5 for CD34^+^ peripheral blood HSPCs. *P < 0.05. CVD: chemical vapor deposition.

We hypothesized that chemical modification of filters may improve this system by increasing the cell recovery and/or delivery efficiency. Fluorinated silanes are advantageous due to their inherent immiscibility with aqueous solutions and propensity to create SLIPS when combined with the appropriate lubricants. With this idea in mind, we developed fluorinated coatings for the porous membranes (Figure 3A). Air plasma activation of the PET filters for 30 s was initially used to introduce reactive oxygen and hydroxyl groups to the surface of the filters to react with the reactive Si–Cl groups of fluorinated silanes. Plasma-treated inserts immediately underwent chemical vapor deposition with trichloro(1*H*,1*H*,2*H*,2*H*-perfluorooctyl)silane (TPFS) for 5–6 h and overnight baking at 65 °C to promote the dehydration reaction that covalently links the silane to the surface. Filter surfaces were then tested for hydrophobicity by contact-angle measurements (Figure S5); treated inserts had significant increases in the water droplet contact angle from 70.3° ± 0.4 to 109.8° ± 0.5 on the top surface of the filter and from 69.9° ± 0.5 to 109.9° ± 0.4 on the bottom surface of the filter, indicating successful silanization. Subsequent infusion with the surface energy-matched fluorinated lubricant perfluoroperhydrophenanthrene (PFPP) created slippery surfaces akin to previously reported SLIPS materials.^43^ Importantly, PFPP is a biocompatible oil used in ophthalmology, making it a suitable choice for biomedical applications. The formation of SLIPS is demonstrated by sliding-angle observations revealing water droplets placed on PFPP-infused fluorosilanized insert slides with minimal tilting and without pinning, which are not observed on inserts infused with oil but not silanized or infused with a silicone-based oil (Movies S1 and S2).

### Testing Cargo Delivery to Model and Primary Cells Using Modified Inserts

To understand the impact that chemical modification of inserts may have on transfection, we tested the delivery of FITC-Dex to Jurkat cells and peripheral blood-mobilized CD34^+^ HSPCs (500,000 cells per experiment). As described previously, cells were subjected to FP, washed, and evaluated for fluorescence under flow cytometry within 1 h after FP. Inserts treated with SLIPS were tested against untreated (FP-Dex), oil-only-treated (FP-Dex-PFPP), and fluorinated silane-only-treated (FP-Dex-Silane) inserts; untreated, Incub-Dex, and FP-Mock were used as controls. Our results showed delivery efficiencies of 61 ± 11% for nontreated inserts, 64 ± 13% for PFPP-only-treated inserts, 74 ± 11% for silane-only-treated inserts, and 64 ± 10% for SLIPS-treated inserts in Jurkat cells. For CD34^+^ HSPCs, we observed delivery efficiencies of 63 ± 11% for nontreated inserts, 65 ± 12% for PFPP-only-treated inserts, 73 ± 8% for silane-only-treated inserts, and 74 ± 10% for SLIPS-treated inserts. In both cell types, control conditions show minimal to no background fluorescence (Figure 3B). Because the same FITC-Dex solution was used for all conditions within each independent experiment, a matched pairwise comparison was chosen for statistical analysis to eliminate potential variability in the cargo concentrations between the multiple independent runs. Statistically significant improvements in efficiencies were observed when nontreated and silane-treated groups were compared in Jurkat cells (*p* value = 0.015). Similar results were observed in CD34^+^ HSPCs with additional significance established between the SLIPS-treated group and nontreated insert groups for this cell type (*p* values of <0.05 for both conditions). Viabilities determined by DAPI staining at the time of flow cytometry shortly after cell manipulation show viabilities in the 60% range for Jurkat cells across treated groups and viabilities between 66 ± 9 and 80 ± 2% for CD34^+^ HSPCs (Figure 3C). Conditions in which cells were filtroporated in the presence of FITC-Dex show decreased viabilities compared to FP-mock controls, indicating compounded cytotoxicity from the treatment and cargo together.

These results demonstrate that filter membrane treatment with the fluorinated silane TPFS is sufficient to increase the delivery efficiency significantly in both Jurkat cells and CD34^+^ HSPCs. We hypothesized that this effect may be due to improved cargo recovery in silane-coated conditions given the hydrophobic nature of these treated inserts, which may, in turn, prevent biomolecular cargoes from adhering to the inset’s surface during FP. To test this hypothesis, we subjected inserts used in FP-Dex delivery experiments to fluorescence microscopy and observed a modest decrease in fluorescence in silane-treated inserts (normalized to nontreated filters; Figure S6). These data are consistent with the hypothesis that less cargo remains on the filter when membranes are made hydrophobic but likely only partly explains the mechanism of this phenomenon, given the modest decrease found in these imaging studies.

### CRISPR/Cas9 Gene Knockout by Filtroporation of Model and Primary Cells

**Figure 4:**
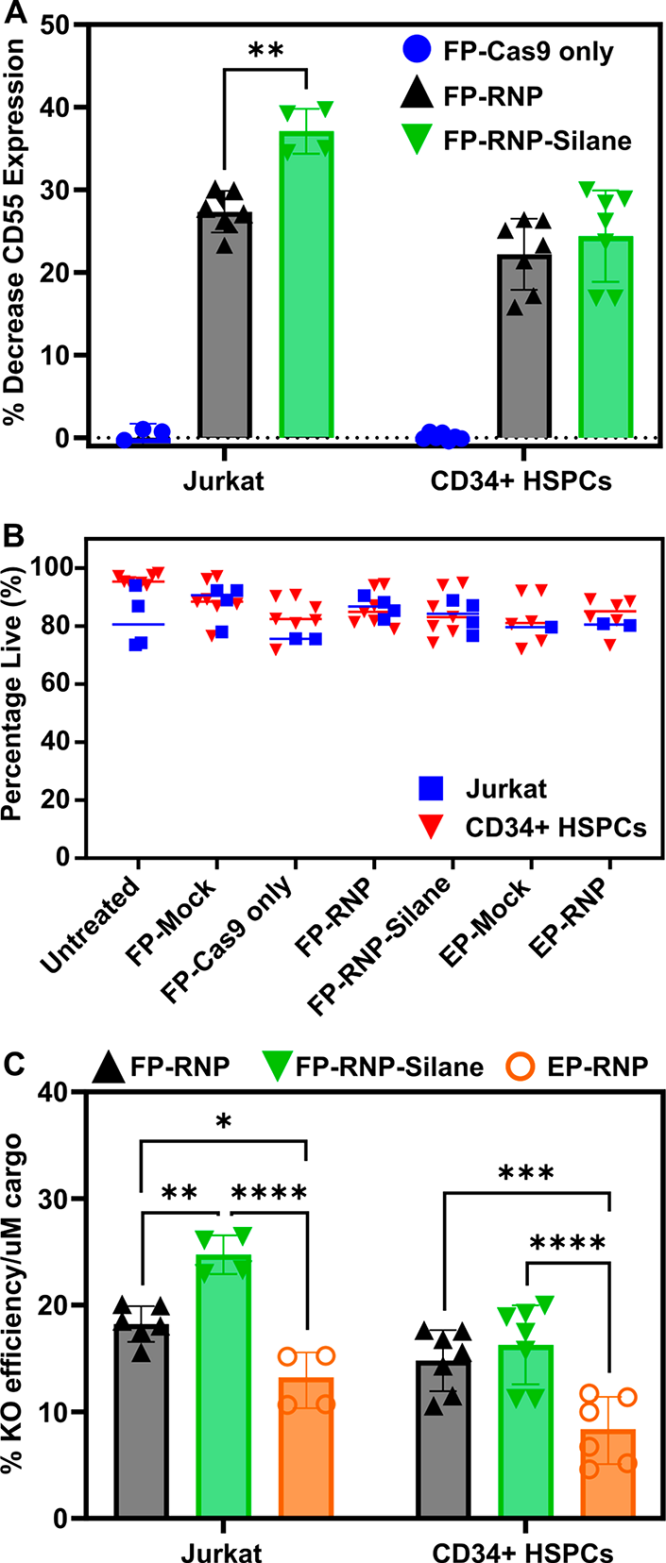
Filtroporation enabling the Cas9 ribonucleoprotein (RNP)-mediated knockout of CD55. (A) Decrease in the CD55 expression compared to Mock controls as determined by flow cytometry of Jurkat cells (96 h) and CD34^+^ hematopoietic stem and progenitor cells (HSPCs) (72 h) after FP. Cells were either filtroporated with Cas9-only without sgRNA (FP-Cas9 only) or treated in the presence of CD55-targeting RNP with nontreated (FP-RNP) or silanized inserts (FP-RNP-Silane). (B) Viabilities determined by DAPI staining at the time of flow cytometry. (C) Comparison of the knockout efficiency per micromolar of RNP cargo between FP (FP-RNP and FP-RNP-Silane) and nucleofection (EP-RNP). *P < 0.05, **P < 0.005, ***P < 0.001, and ****P < 0.0001.

To test whether our system was capable of performing the delivery of clinically relevant cargo to target cells, we sought to deliver Cas9 RNPs by FP. We targeted the surface-expressed protein CD55, present on all blood cells,^46,47^ and evaluated the CD55 expression by flow cytometry and sequencing as a surrogate for our system’s delivery efficiency. A concentration of 300 pmol of Cas9 protein was found to be optimal in yielding robust knockout efficiencies while preventing overt cytotoxicity associated with cargo and cell manipulation. The final concentration of RNP in solution was 1.5 μM (as a comparison, the final concentration of 5 μM was used in control nucleofection experiments, while previous FP reports used 25 μM or 17 times more). Flow cytometry results reveal 27 ± 3% knockout of CD55 for nontreated inserts in Jurkat cells at 96 h after filtroporation (FP-RNP). This increased to 37 ± 3% (*p* value = 0.0035) in silane-treated groups (FP-RNP-Silane; Figure 4A). For CD34^+^ HSPCs, 22 ± 4% and 24 ± 6% of cells had CD55 knocked out in nontreated and silane-treated conditions, respectively (Figure 4A), under flow cytometry analysis at 72 h after filtroporation (see the gating strategy and representative flow cytometry plots in Figures S7 and S8). As a control, cells were also filtroporated with Cas9 only without synthetic single-guide RNA (sgRNA; FP-Cas9 only), and no impact on the CD55 expression was observed, indicating that decreases in the expression are the result of true gene knockout. Sanger sequencing was performed on polymerase chain reaction (PCR)-amplified extracted genomic DNA, followed by the tracking of insertions and deletions (INDELs) by decomposition (TIDE) or inference of CRISPR edits (ICE) analyses to test whether the cellular genome had been modified in these experiments (Figure S9A). These sequencing results reveal genomic INDELs at the CD55 locus that match flow-cytometry results, confirming RNP-mediated genomic knockout.

Jurkat recovery immediately following transfection was variable due to the tendency of Jurkat cells to form clumps. Cell counting by TB counterstaining revealed clumps that appear to shear as they are pulled through the pores, underscoring the importance of careful cell suspension preparation and clump separation to maximize cell recovery. Live cell recovery (estimated as the fraction of live cells counted after treatment divided by the number of live cells in the untreated group) ranged from 16 ± 6% to 22 ± 5% with no statistically significant difference between the groups (Figure S9B). Recovery of CD34^+^ HSPCs was consistently higher at between 52 and 81% across conditions (Figure S9C). The recovery and total live cell number at 24 h were also reported and indicate cell loss during recovery from treatment because some cells undergo apoptosis within the first day after FP (Figure S9D,E). Viabilities at 72 h (HSPCs) and 96 h (Jurkat cells) were greater than 80% across all treated groups (Figure 4B), indicating that cells can recover after FP. Importantly, these viabilities are similar to those observed in electroporation experiments. Both Jurkat cells and HSPCs proliferated at comparable rates and similar to nucleofection controls while in culture after FP (Figure S9F,G), indicating that FP does not damage or disrupt cells’ abilities to divide.

### Comparison to Electroporation

To determine whether our platform can perform at efficiencies comparable to those of commercially available technologies such as nucleofection, control experiments were conducted in parallel to FP. A typical RNP concentration of 5 μM for nucleofection was used in these experiments. Given the larger volume of solution in FP (200 μL), it was not cost-effective to match the cargo concentration of the FP system with that of electroporation because this would require large amounts of Cas9 and sgRNA. Reports in the literature suggest that the knockout efficiency by electroporation increases linearly as the cargo concentration is increased.^48,49^ Therefore, to compare the two methodologies, we compared the KO efficiency per unit of cargo in solution for both experimental techniques. Calculations indicate that FP enables significant improvements in KO efficiency/μM of cargo (Jurkats, 18 ± 2%/μM for nontreated and 25 ± 2%/μM for silanized inserts; HSPCs, 15 ± 3%/μM for nontreated and 16 ± 4%/μM for silanized inserts) compared to nucleofection (13 ± 3%/μM for Jurkats and 8 ± 3%/μM for HSPCs), with statistically significant differences in both nontreated and silane-treated filter conditions for Jurkat cells (*p* values of <0.005 and <0.0001, respectively) and for CD34^+^ HSPCs (*p* values of 0.001 and <0.0001, respectively) (Figure 4C).

## Conclusions and Prospects

We developed and demonstrated a FP-based cell deformation platform for the intracellular delivery of fluorescently labeled dextran, plasmids, and Cas9 RNPs assembled from common laboratory equipment with the goal of democratizing access to cell transfection. This platform was shown to modify Jurkat and CD34^+^ HSPCs genetically in a format that costs <$10 in materials per experiment. This method will enable more laboratories around the world to engage in research because high costs associated with cargo delivery platforms is a barrier to entry for researchers with limited resources. Importantly, our devices outperform electroporation efficiencies in terms of knockout percentage per unit of cargo. Further studies are required to evaluate the retainment of stemness and engraftment potential in treated HSPC populations. However, our method is promising given that other similar FP and mechanoporation methodologies reported do not alter the stem-cell differentiation potential, as determined by engraftment studies.^42^ Efforts are currently underway to elucidate the mechanisms underpinning membrane repair following mechanical disruption, as well as the impact of our permeabilization methodology on cellular activity and transcriptomic profiles via RNA-Seq. Further studies are required to understand the mechanism behind fluorinated silane-mediated increases in the delivery efficiency, and its apparent cell- and cargo-type dependences. One limitation of this approach is that commercially available cell culture inserts are only manufactured with predetermined pore sizes and possess random pore distribution due to the nature of the manufacturing technique. The 8 μm filters used here were suitable for transfection of Jurkat cells and HSPCs but were not suitable for delivery to human primary T cells or K562 cells due to size limitations. Manufacturing membranes with selected pore size and distribution would enable applications of this platform to any cell type and are thus a promising strategy to universalize FP approaches to cell transfection. Understanding the mechanism of cell poration and strategies that may facilitate this technique may prove important in boosting delivery efficiencies further.

## Materials and Methods

### Device Fabrication and Operation

Two 15 mL conical tubes (Corning) were punctured with a 20 gauge (BD Precision Guide) needle below the 1 mL mark, such that the perforation of the top tube (tube A as described) fits within the opening of tube B after insertion (Figure 1A–C). A flexible transparent piece of tubing was used to connect the perforation in tube B with the vacuum outlet of the biosafety cabinet. The vacuum was measured with a pressure gauge and consistently measured −20″ Hg. For parallelized system operation with 3D-printed chambers, 6 × 15 mL conical tubes each with a perforation below the 1 mL line were placed into the slots, uncapped and loaded with one insert each. After the cell suspension was added to the insets (12-well PET; catalog 353182, Falcon Corning), the vacuum line was connected to the chamber and applied to the system. The postfiltroporation cell suspension was collected and transferred to cell culture plates for culture or processing.

### 3D Printing

Chambers were constructed by using a stereolithographic 3D printer (Form 3, Formlabs) with flexible V2 resin (FLFLGR02, Formlabs). Chambers were designed using 3D modeling software, printed, washed in isopropyl alcohol (Formlabs Wash chamber), dried with nitrogen gas, and postcured (Formlabs Cure chamber) at 60 °C for 15 min. After this postcuring process, supports were removed by hand from the print.

### Filter Surface Functionalization

Filters were first air-plasma-treated at 100 W and 8 standard cubic centimeters per minute of air (HPT-200, Henniker Plasma) for 30 s and immediately transferred to a desiccator with 200 μL of TPFS (Sigma-Aldrich) placed on a glass slide. After negative pressure was established in the desiccator, the vacuum was turned off and the inserts remained under vacuum for 5–6 h. The inserts were then transferred to an oven (20E Lab Oven, Quincy Laboratories) at 65 °C overnight. Prior to use in experiments, 5 μL of water was pipetted onto the filters to ensure the success of functionalization by verifying that the surface was rendered hydrophobic.

### Filter Characterization

To test surface functionalization with TPFS, filters were cut from the inserts, and water droplets were placed on the surface and imaged using an FTA1000 drop shape instrument contact-angle goniometer. Measurements and analyses of the contact angles were made by using *ImageJ* software.

### Jurkat and CD34^+^ Hematopoietic Stem and Progenitor Cell Cell Culture

Jurkat cells (American Type Culture Collection, Inc., ATCC) were cultured in 1× RPMI 1640 with l-glutamine supplemented with 10% fetal bovine serum (FBS; Gibco) and 1% penicillin/streptomycin (10,000 units/mL penicillin and 10 mg/mL streptomycin) (Gibco). Peripheral blood CD34^+^ HSPCs were purchased from STEMCELL Technologies after mobilization by either granulocyte colony-stimulating factor (G-CSF) alone or G-CSF and Plerixafor. Cells were thawed and prestimulated as described by Hoban et al.^50^ Briefly, cells were thawed in Iscove’s modified Dulbecco’s medium (IMDM; Gibco) containing 20% FBS and then prestimulated for 24 h in prestimulation media composed of StemSpan Serum-Free Expansion Medium II (SFEM-II; STEMCELL Technologies) supplemented with penicillin/streptomycin/glutamine (P/S/Glu) (diluted 100× for final concentration; Thermo Fisher Scientific) and recombinant human stem cell factor (rhSCF), human thrombopoietin (Tpo), and recombinant human Flt3-ligand (Flt3-L) (all cytokines from Peprotech) to a final concentration of 50 ng/mL. Cells were treated by FP in this medium and transferred to basal bone marrow medium (BBMM) at 24 h after transfection. The BBMM is composed of IMDM plus 20% FBS, P/S/Glu 100× diluted, and 0.5% bovine serum albumin (Millipore Sigma) and supplemented with recombinant human stem cell factor, recombinant human interleukin-3 and interleukin-6 (Peprotech) to a final concentration of 50 ng/mL.

### FITC-Dex, Plasmid, and RNP Delivery

The desired numbers of cells (500,000 for CD34^+^ HSPCs and 500,000 to 4 million for Jurkat cells) were centrifuged and resuspended in either a regular medium or a cargo-containing medium. For untreated and mock samples, 200 μL of FBS-free RPMI (Jurkat cells) or a prestimulation medium (HSPCs) was used to resuspend cells.

For FITC-Dex-treated samples, cells were resuspended in the same volume of the respective cell media containing 300 μg/mL of FITC-Dex (40 kDa, Millipore Sigma). Cells were collected after FP, washed at least once with fresh 1× PBS, and resuspended in 300 μL of PBS for flow cytometry.

For eGFP-plasmid-treated samples (eGFP expression vector, Plasmid 11153, Addgene), Jurkat cells were resuspended in the same final reaction volume of 200 μL containing the 0.1 mg/mL plasmid. After treatment, cells were transferred to cell culture plates and a complete RPMI medium was added for a final concentration of 700,000 cells/mL. Cells were incubated at 37 °C and monitored over 24–72 h by daily TB counterstaining cell counts (Countess, Thermo Fisher Scientific). Each day, samples of at least 100,000 cells were obtained and fixed in 0.5% paraformaldehyde in PBS for downstream flow-cytometry analysis, and at least 400,000 cells were used for mRNA extraction, purification (RNeasy Plus Mini Kit, Qiagen), reverse transcription (M-MLV reverse transcriptase, Thermo Fisher Scientific), and ddPCR (QX200 ddPCR System, Bio-Rad). See the Additional Information on RNA Extraction and ddPCR section for further details.

For Cas9 RNP experiments, RNPs were prepared by mixing 300 pmol of Cas9 (Macrolab) with 360 pmol of synthetic modified sgRNA (Synthego) (in a 1:1.2 ratio) and incubating on ice for 10 min. RNPs were then added to the appropriate cell media for Jurkat cells or HSPCs for a total volume of 200 μL. Post-treatment cells were placed in wells of cell culture plates, and a fresh complete medium was added to reach a final concentration of 500,000 cells/mL (CD34^+^ HSPCs and Jurkat cells). At 24 h, the cell viability was measured by TB counterstaining for both cell types. For CD34^+^ HSPCs, cells were centrifuged at low speed (100*g* for 10 min) to remove dead cells and transferred to BBMM at a final concentration of 100,000 cells/mL for the next 48 h prior to staining and flow cytometry.

### Nucleofection

RNPs were prepared as previously described at 100 pmol of Cas9 with 120 pmol of CD55-targeting sgRNA. A total of 200,000 Jurkat cells or CD34^+^ HSPCs per condition were pelleted at 90*g* for 15 min and resuspended in 20 μL of nucleofection buffer (P3 Primary Cell 4D-Nucleofector X Kit for stem cells and SE Cell Line 4D Kit for Jurkat cells; Lonza) with or without RNPs. Cells were transferred to a well in a 16-well strip, allowed to settle for 10 min, and placed in a 4D-Nucleofector (Lonza). The program CL-120 was used for Jurkat cells, and the program DZ-100 was used for HSPCs. After treatment, cells were allowed to rest for another 10 min, and then 80 μL of the appropriate cell medium was added to the strip well, and the entire volume transferred to a well in a cell culture plate containing a cell medium for a final cell concentration of 400,000 cells/mL. Plates were incubated at 37 °C for the following 72–96 h.

### Fluorescence Microscopy

Cells were prepared in cell culture plates by fixing with 0.5% paraformaldehyde (Sigma-Aldrich) overnight at 4 °C in the dark. Nuclear stain DAPI (Thermo Fisher Scientific) was diluted to a working solution concentration of 1 μg/mL and added to the sample for a final concentration of 0.05 μg/mL. Fluorescence microscopy images were taken on an EVOS M5000 instrument (Invitrogen, Thermo Fisher Scientific).

### Scanning Eelectron Microscopy

Silicon oxide chips were spin-coated with Gorilla Glue at 5000 rpm by using a G3P-8 spin coater (Specialty Coating Systems). The glue was allowed to cure for 1 h at room temperature, while cells were fixed in 3% glutaraldehyde (Sigma-Aldrich) in a 1× PBS solution. After 15 min of fixation, cells were placed on silicon chips and allowed to rest for 5 min to enable contact between the cells and glue to take place. It was critical from this step forward that chips were never allowed to dry completely or cell deformation would occur; all solutions were prewarmed to 37 °C. Preparation proceeded as described by previously published protocols.^51^ Briefly, chips were transferred to an appropriately sized cell culture plate well and washed three times over 15 min with 1× PBS. Chips were transferred to a fresh well, and a 1% osmium tetroxide (Millipore Sigma) solution was added to cover the surface. Incubation took place over 20 min, while the container was covered to avoid evaporation. The sample was then washed five times with 1× PBS over 10 min and incubated in freshly made 1% carbohydrazine (Sigma-Aldrich) in a water solution for 20 min. The sample was washed with distilled water five times over 15 min and incubated again in a 1% osmium tetroxide solution as previously described for 20 min. From this point on, solutions were kept at room temperature, but care was still taken to avoid sample drying. Samples were rinsed three times with distilled water over 15 min and placed in another dish. Sequential dehydration with ethanol was then performed by incubating samples in increasing concentrations of ethanol in water (30, 50, 70, 90, and 100%) over 30 min (6 min each step). Samples were dried in a critical point dryer (Tousimis Autosamdri-810 Critical Point Dryer) and sputtered with 1–2 nm of gold for electron microscopy. Samples were mounted on studs and imaged using a Zeiss Supra 40 V scaning electron microscope under a vacuum at a voltage of 3 kV.

### Antibody Staining

Anti-CD55 antibody (APC antihuman CD55 Mouse Monoclonal Antibody, BioLegend) was diluted 100× in a cell staining buffer (SouthernBiotech). Up to 1 million Jurkat cells or CD34^+^ HSPCs were centrifuged at 500*g* for 5 min and resuspended in 100 μL of a diluted antibody solution. The cell suspension was incubated in the dark at 4 °C for at least 30 min and subsequently washed twice with a cell staining buffer. Cells were then resuspended in 300 μL of a cell staining buffer, kept on ice, and taken to flow cytometry within 1 h.

### Flow Cytometry

Flow-cytometry data were acquired and processed using an LSR Fortessa cytometer (BD Biosciences). Data were analyzed by using *FlowJo* software (FlowJo, LLC).

### DNA Extraction and Sequencing

After at least 48 h postdelivery of RNPs, DNA from at least 100,000 cells was extracted using QuickExtract (QE) DNA Extraction Solution (Lucigen). Cells were centrifuged and resuspended in 1 μL of QE for every 10,000 cells. The cell suspension was then placed in a thermocycler (65 °C for 20 min, 95 °C for 10 min, and 8 °C for infinity), and DNA was ready for downstream use. For PCR, DNA was diluted 10× in molecular-biology-grade water and 1–20 μL used in protocols. DNA was amplified with primers specific from the region flanking the sgRNA cut site (forward primer 5′-CCCGTCTTGTTTGTCCCACC-3′ and reverse primer 5′-AGACACAAGCCCCCTTGAAA-3′; Integrated DNA Technologies) using Platinum SuperFi II Polymerase Master Mix (Thermo Fisher Scientific), run on a 2% agarose gel to check for a single band, and submitted for Sanger sequencing (Laragen). The estimation of INDELs was performed using the TIDE analysis tool or Synthego’s ICE tool.

### Additional Information on RNA Extraction and ddPCR

ddPCR was used to measure the mRNA levels of the transfected cells. Detection of mRNA was used as a tertiary assay to assess whether the DNA was delivered to the nuclei of the cells. Using ddPCR, we confirmed the successful delivery of the GFP plasmids to Jurkat cells (Figure S2C). We observed significant differences in the copy/μL values compared to the negative controls, which ran the cells through the vacuum filter membrane without any added DNA (Figure S2C).

Extraction of RNA and reverse transcription were first performed before ddPCR after collecting cells. First, ∼5 × 10^5^ cells were pelleted and resuspended in 100 μL of lyses buffer from RNeasy Plus Mini Kit (Qiagen). Total RNA was extracted from collecting cells with spin columns (RNeasy Plus Mini Kit; Qiagen) and followed the manufacturer’s protocol. The RNA quality was determined by using a NanoDrop spectrophotometer (Thermo Fisher Scientific). All of the RNA samples used for the study were pure (*A*_260_/*A*_280_ ≥ 1.9; *A*_260_/*A*_230_ ≥ 2). Then, 200 ng of RNA was subjected to reverse transcription in 50 μL of reaction using M-MLV reverse transcriptase (Thermo Fisher Scientific) and random hexamers (Thermo Fisher Scientific). The reactions were carried out at 37 °C for 50 min and stopped by incubation at 70 °C for 15 min.

ddPCR was performed with a QX200 ddPCR System (Bio-Rad), according to the manufacturer’s protocol. Briefly, each of the 20 μL reactions contained 1× EvaGreen ddPCR Supermix (Bio-Rad), 250 nM gene-specific primers, and 2 μL of the cDNA sample. The following primers for CD19RCD28MZ were designed with Vector NTI software: forward, 5′- CCTGGTGAAGGGCTTCTACC-3′; reverse, 5′- CGGAGCAGCTAAAGACGTTG-3′ (179 bp amplicon). Primers targeting GFP were designed based on work previously reported.^52^ Human beta actin (SKU 10031258) primers were used as the internal control (Bio-Rad). Each reaction was mixed with 70 μL of Droplet Generation Oil (Bio-Rad), partitioned into 14,000–17,000 droplets in a QX200 Droplet Generator (Bio-Rad), transferred to 96-well plates (Bio-Rad) and heat-sealed with foil by a PXTM PCR Plate Sealer (Bio-Rad). The PCR reactions were performed in a T100TM Thermal Cycler (Bio-Rad) with the following cycling conditions: 1× (95 °C for 5 min), 40× (95 °C for 30 s and 60 °C for 1 min), 1× (4 °C for 5 min and 90 °C for 5 min) with a 2 °C/s ramp rate and held at 4 °C. Immediately following end-point amplification, the fluorescence intensity of individual droplets was measured with a QX200 Droplet Reader (Bio-Rad). After data acquisition, data analyses were performed with *QuantaSoft* droplet reader software (Bio-Rad). The absolute transcript levels reported are copies/μL of the final 1× ddPCR reaction.

### Statistical and Image Analyses

One-way and two-way ANOVA analyses were performed using *GraphPad Prism 9* software. Quantification of the filter surface fluorescence was performed using *ImageJ* software.

## Supporting Information Available

Supplementary figures including a photograph of the vacuum chamber, SEM of filters, flow-cytometry plots of delivery experiments, results of ddPCR assays, contact-angle experiments, fluorescent microscopy, and quantification of membrane fluorescence, cell recovery, viability, and growth (PDF)
Video of water droplets added to the surfaces of filters treated with PFPP oil only, without silanization (MP4)
Video of water droplets added to surfaces treated with fluorosilane and later lubricated with PFPP oil (creating a slippery liquid-infused porous surface) (MP4)
