# Supplementary material for: Fluorinated Silane-Modified Filtroporation Devices Enable Gene Knockout in Human Hematopoietic Stem and Progenitor Cells
Source: ACS Appl Mater Interfaces. 2023 Aug 24;15(35):41299–309. doi: 10.1021/acsami.3c07045 (PMC10485797; doi:10.1021/acsami.3c07045)
Supplement: Supplementary file 1 — am3c07045_si_001.pdf [file am3c07045_si_001.pdf]

Supporting Information for

Fluorinated Silane-Modified Filtroporation Devices Enable Gene Knockout in Human Hematopoietic Stem and Progenitor Cells

Isaura M. Frost[a,b,c], Alexandra M. Mendoza[d,e], Tzu-Ting Chiou[c], Philseok Kim[f], Joanna Aizenberg[f], Donald B. Kohn[g,h,i], Satiro N. De Oliveira[c], Paul S. Weiss*[a,d,e,j], and Steven J. Jonas*[c,e,i,k]

[a]Department of Bioengineering, University of California, Los Angeles, Los Angeles, California 90095, United States

[b]UCLA Medical Scientist Training Program, David Geffen School of Medicine, University of California, Los Angeles, Los Angeles, California 90095, United States

[c]Department of Pediatrics, David Geffen School of Medicine, University of California, Los Angeles, Los Angeles, California 90095, United States

[d]Department of Chemistry and Biochemistry, University of California, Los Angeles, Los Angeles, California 90095, United States

[e]California NanoSystems Institute, University of California, Los Angeles, Los Angeles, California 90095, United States

[f]Department of Chemistry and Chemical Biology, Harvard University, Cambridge, Massachusetts 02138, United States

[g]Department of Molecular and Medical Pharmacology, University of California, Los Angeles, Los Angeles, California 90095, United States

[h]Department of Microbiology, Immunology and Molecular Genetics, University of California, Los Angeles, Los Angeles, CA 90095;

[i]Eli & Edythe Broad Center of Regenerative Medicine and Stem Cell Research, University of California, Los Angeles, Los Angeles, CA 90095;

[j]Department of Materials Science and Engineering, University of California, Los Angeles, Los Angeles, CA 90095;

[k]Children's Discovery and Innovation Institute, University of California, Los Angeles, Los Angeles, CA 90095

*To whom correspondence should be addressed. Email: psw@cnsi.ucla.edu (P.S.W.) and sjonas@ucla.edu (S.J.J.).

Supplementary Figures

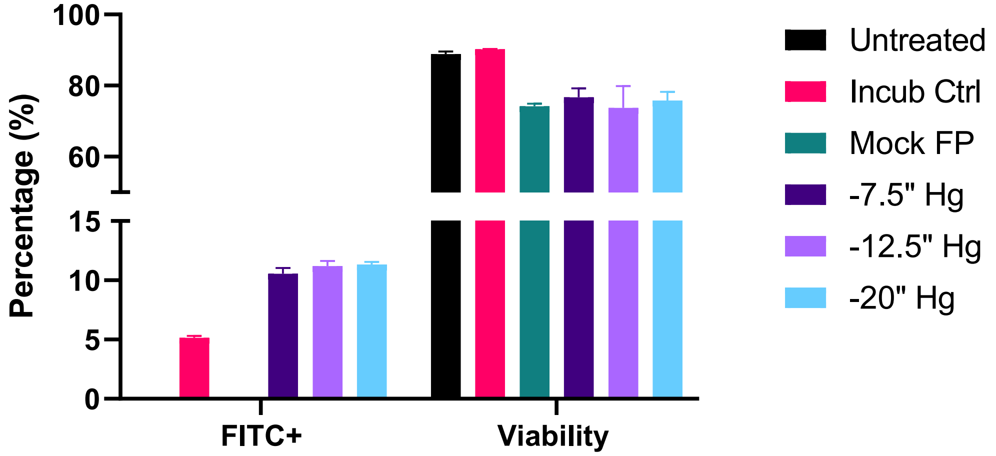

Figure S1. Filtroporation (FP) of human primary T cells with fluorescein isothiocyanate (FITC)-Dextran cargo at various vacuum pressures. (Left) Delivery efficiency determined by flow cytometry and (Right) viability by 4',6-diamino-2-phenylindole (DAPI) nuclear counterstain. Incub Ctrl: cells incubated in FITC-Dextran, but not filtroporated; Mock FP: FP without cargo.

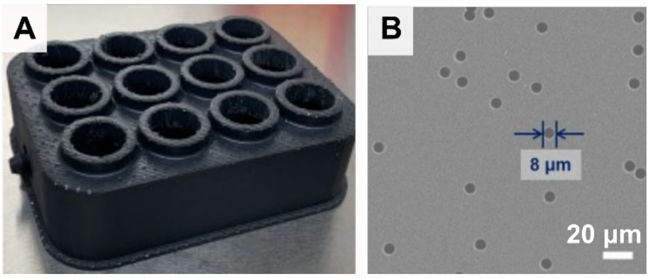

Figure S2. (A) Digital photograph of a 12 slot 3D-printed chamber for multiplexed filtroporation. (B) Scanning electron micrograph of porous filters showing pore distribution and diameter of 8 μm. (Photo credit: Isaura Frost)

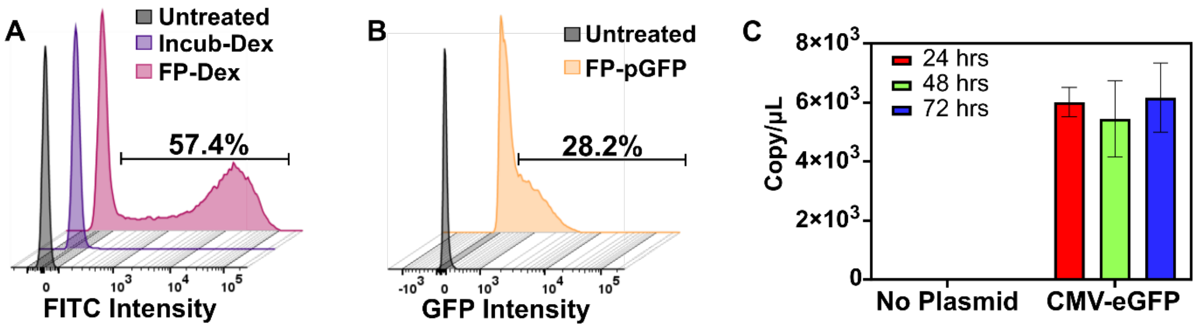

Figure S3. (A) Representative flow cytometry histograms for fluorescently tagged dextran (FITC-Dex) experiments (2 h post-treatment) or (B) green fluorescent protein (GFP)-encoding plasmid experiments to Jurkat cells (72 h post-treatment). Cells were either untreated, incubated with FITC-Dex (Incub-Dex), or treated by filtroporation in the presence of FITC-Dex (FP-Dex). For plasmid experiments, untreated cells were compared to those treated in the presence of GFP plasmids (FP-pGFP). (C) Copy number determined by droplet digital PCR of reverse transcribed GFP mRNA extracts. FITC: fluorescein isothiocyanate; Dex: dextran; Incub: incubation; FP: filtroporation; pGFP: plasmid encoding GFP; eGFP: enhanced green fluorescent protein.

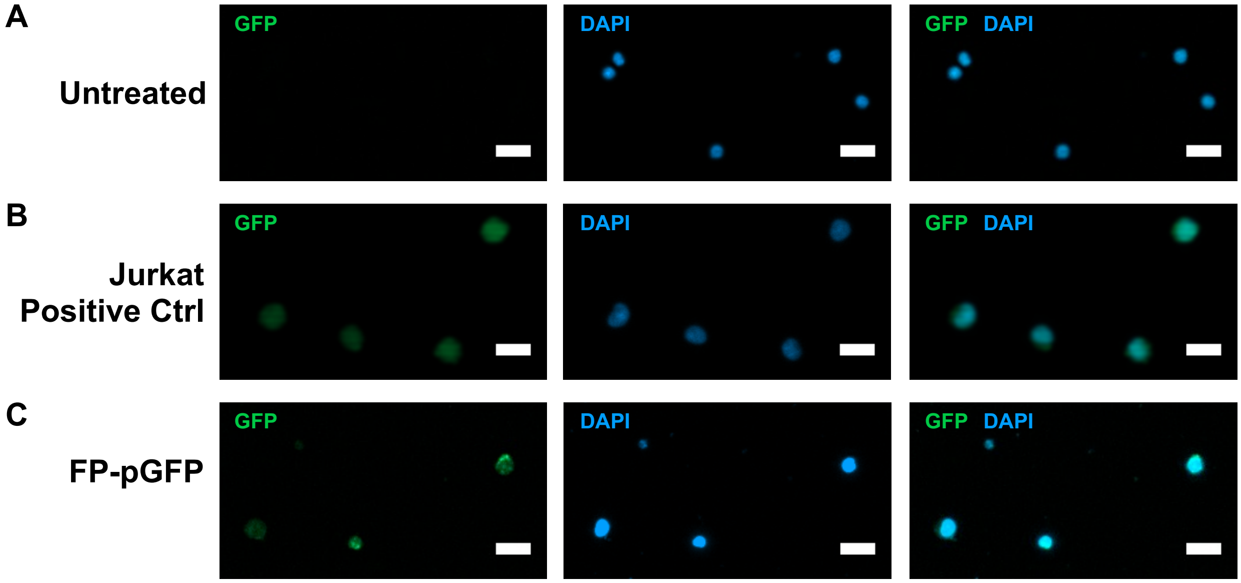

Figure S4. Confocal images of Jurkat cells after filtroporation with green fluorescent protein (GFP)-encoding plasmids (pGFP). Images display GFP channel (left) and 4’,6-diamino-2-phenylindole (DAPI) nuclear counterstain (middle) channels as well as their merge (right). (A) Jurkat wild-type (WT) cells, (B) Jurkat enhanced GFP-epidermal growth factor receptor cell line as a positive control, and (C) CMV (cytomegalovirus) -EGFP transfected Jurkat cells 24 h post-cell deformation. Jurkat cells were incubated with 0.1 mg/mL of the CMV-EGFP plasmid. Scale bars = 20 μm.

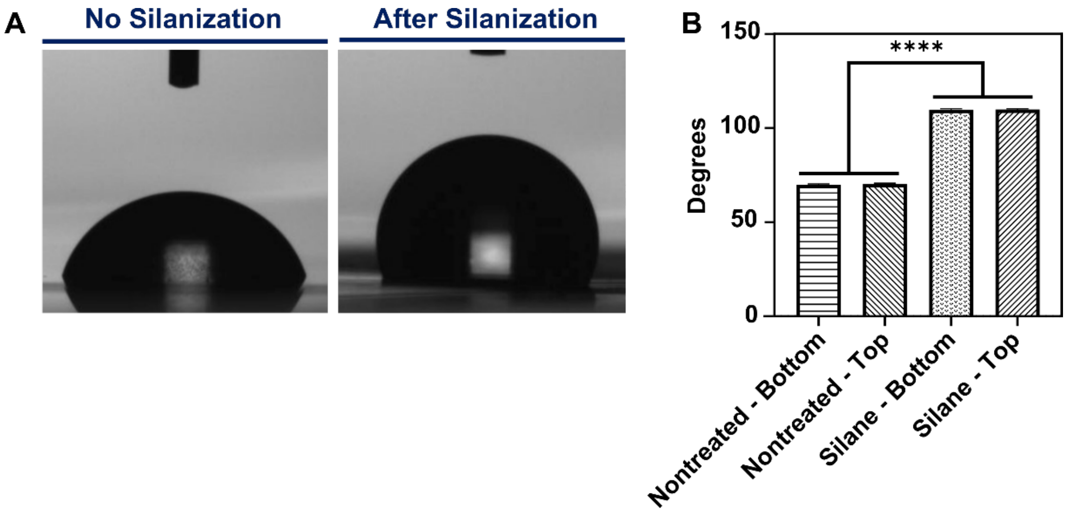

Figure S5. (A) Images of water droplets placed on filter surfaces before and after fluorosilane treatment in contact angle experiments. (B) Quantification of water contact angle performed on top or bottom side of filters cut out from inserts before and after silanization. (****P < 0.0001)

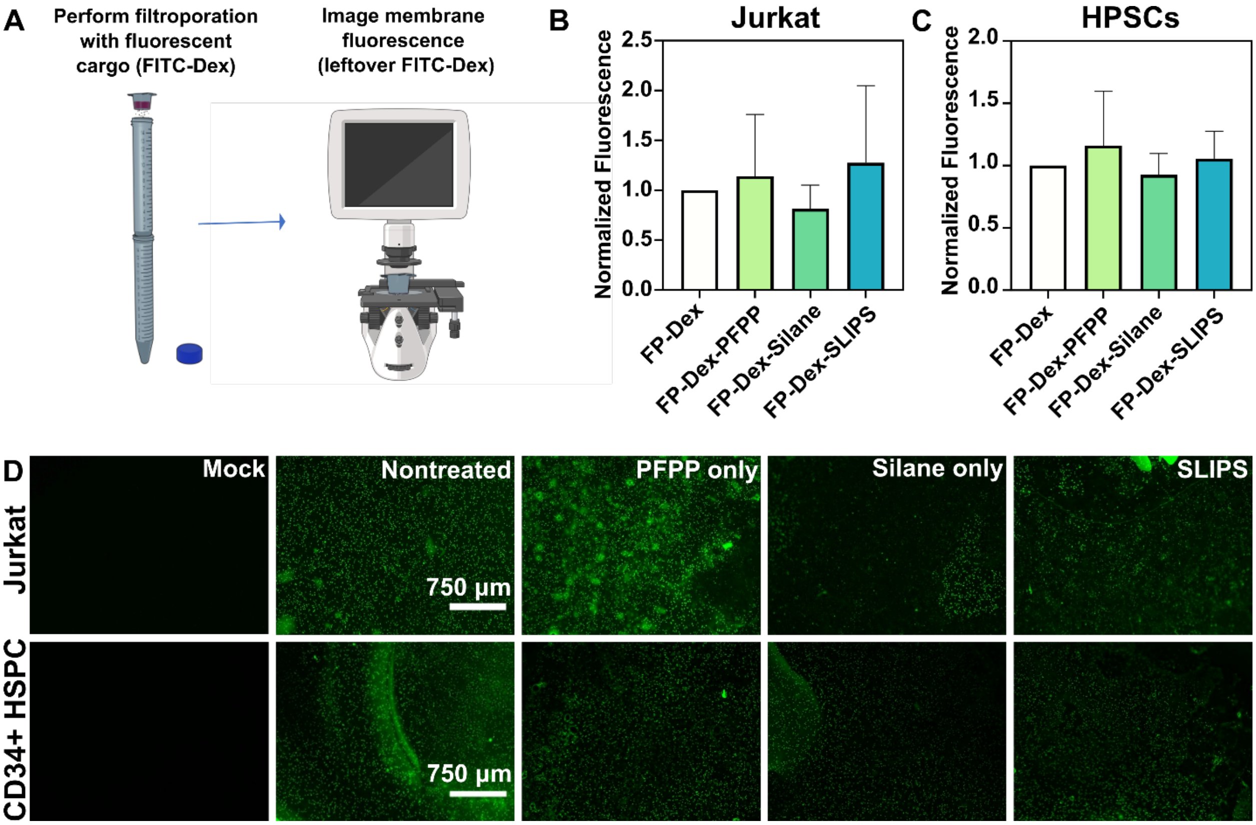

Figure S6. (A) Schematic of fluorescence assessment performed on filters after filtroporation. (B) Quantification by ImageJ software of overall fluorescence of inserts after fluorescein isothiocyanate tagged dextran (FITC-Dex) delivery experiments under different conditions for Jurkat cells (N = 3 independent experiments) and (C) human CD34$^+$ hematopoietic stem and progenitor cells (HSPCs) (N = 4 independent experiments for HSPCs). (D) Fluorescence microscopy images of filters after FITC-Dex delivery for different insert treatments; fluorescent dots represent pores stained with fluorescent cargo, not cells. FP: filtroporation; PFPP: perfluoroperhydrophenanthrene; SLIPS: slippery liquid-infused porous surfaces. Schematic created with BioRender.com.

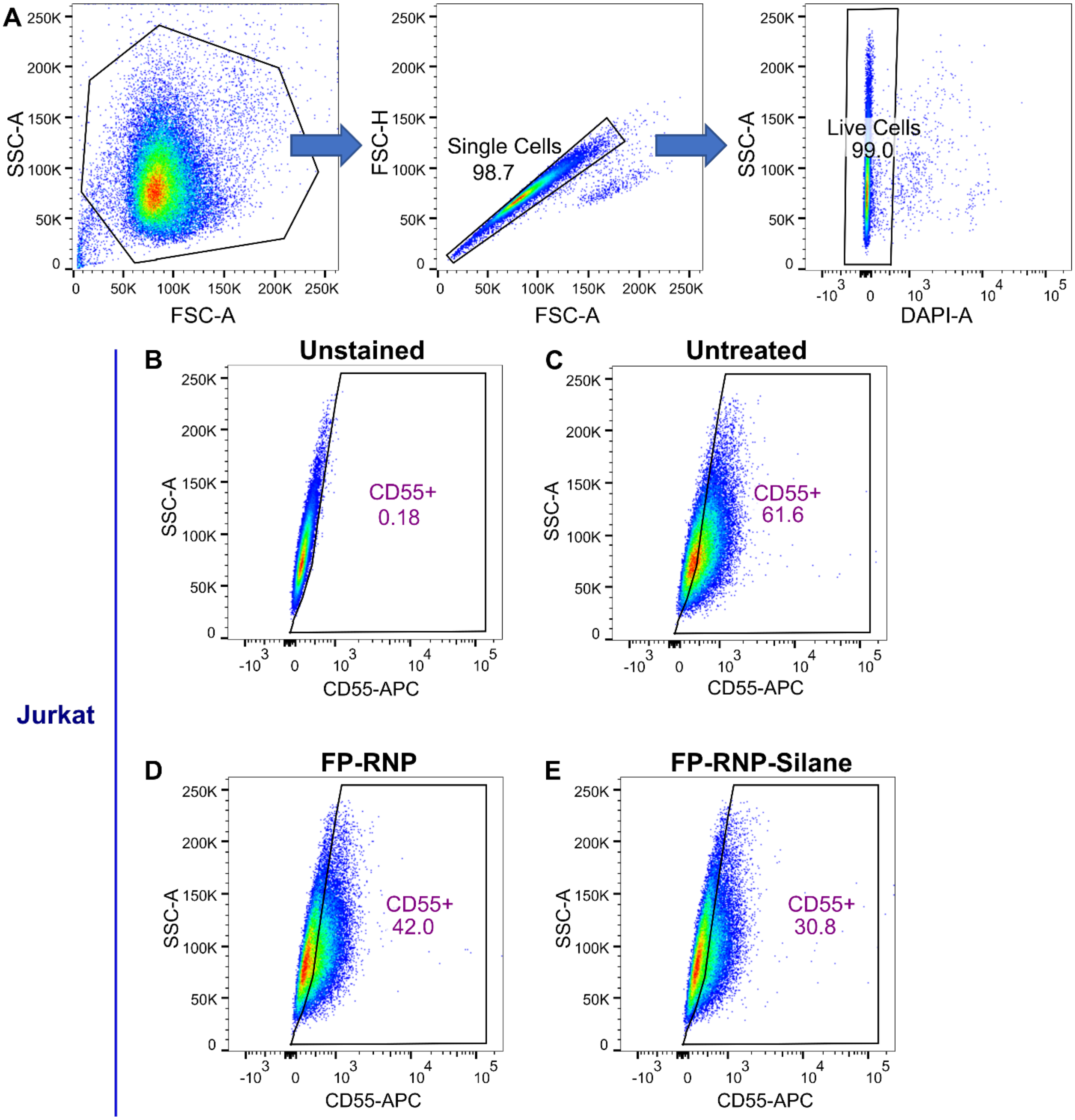

Figure S7. Representative flow cytometry plots showing gating strategy for Jurkat cells. (A) First, cells are selected on forward and side scatter plots, then singlets are selected, and finally dead cells are excluded by 4’,6-diamidino-2-phenylindole (DAPI) staining. For CD55 experiments in Jurkat cells, gating strategy is shown for (B) untreated unstained cells, (C) untreated stained cells (controls), and (D) filtroporation with ribonucleoprotein (RNP) targeting CD55 showing decreased expression in nontreated (filtroporation with RNP (FP-RNP)) and (E) silane-treated inserts (FP-RNP-Silane). A similar gating strategy was used for experiments in human $CD34^+$ hematopoietic stem and progenitor cells (HSPCs). SSC: side scatter; FSC: forward scatter; APC: allophycocyanin.

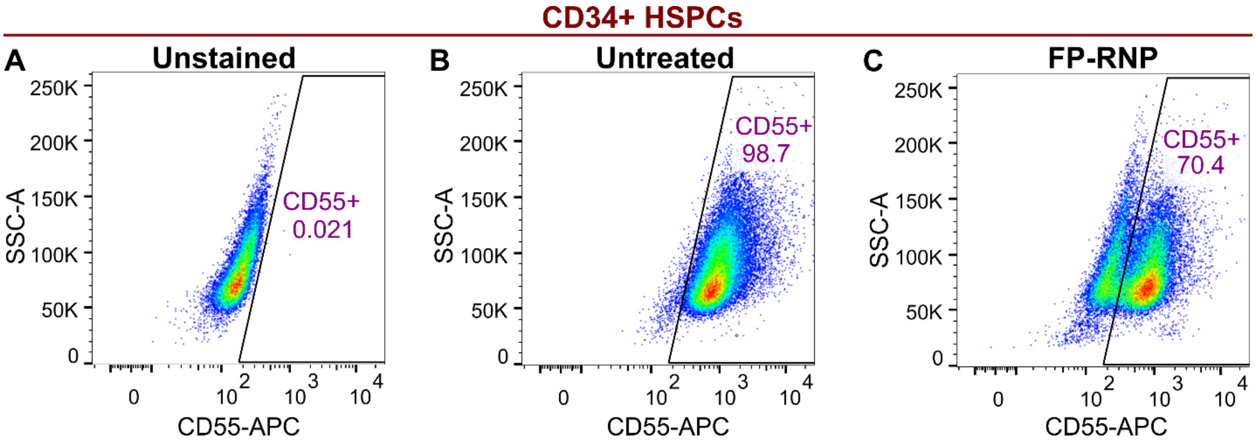

Figure S8. Representative flow cytometry plots showing gating strategy for human CD34$^+$ hematopoietic stem and progenitor cells (HSPCs). For CD55 experiments in HSPCs, gating strategy is shown for (A) untreated unstained cells, (B) untreated stained cells (controls), and (C) filtroporation with ribonucleoprotein (RNP) targeting CD55 showing decreased expression in nontreated (filtroporation with RNP, FP-RNP). SSC: side scatter; FSC: forward scatter; APC: allophycocyanin.

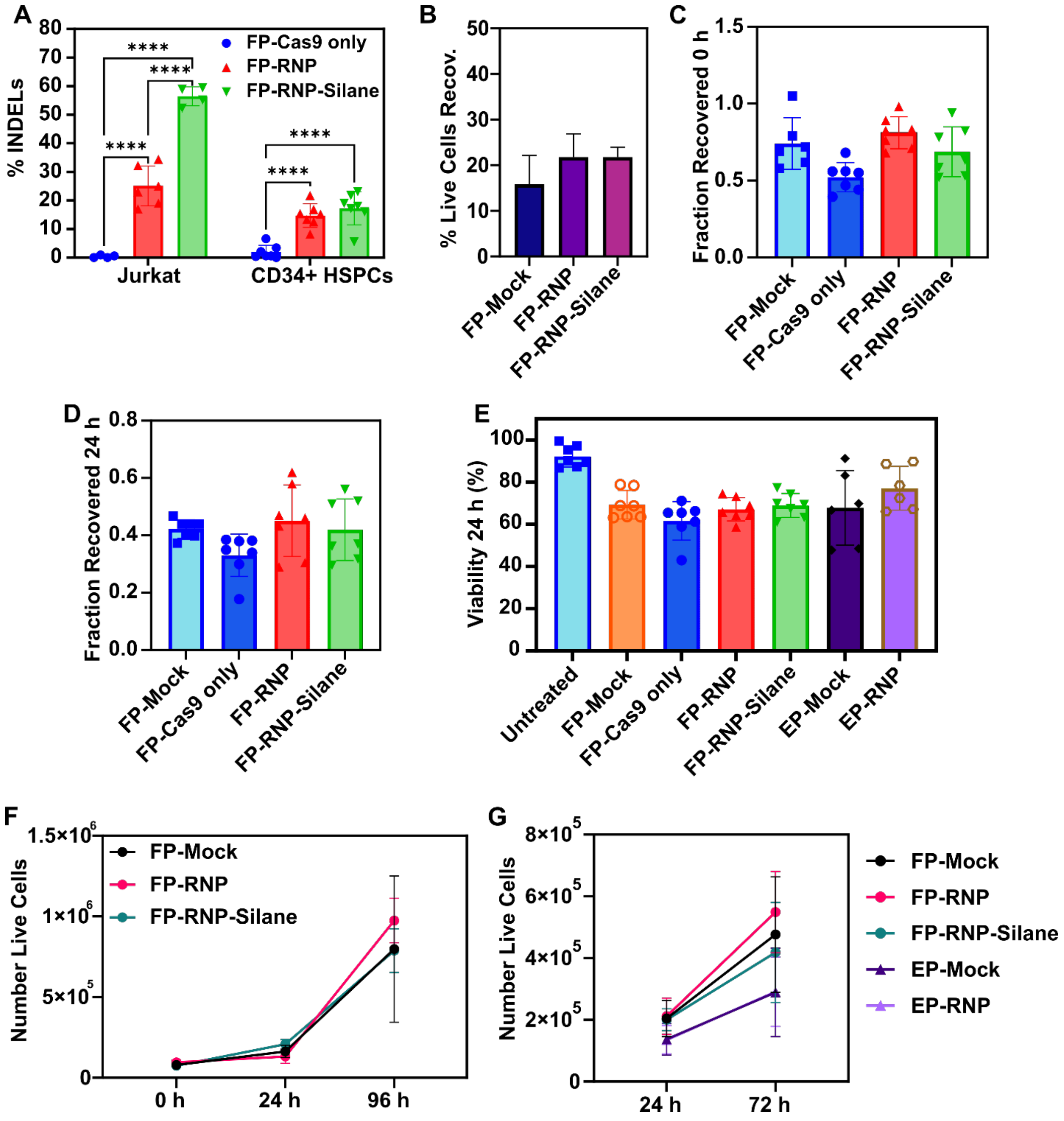

Figure S9. (A) Insertions and deletions (INDELs) determined after filtroporation (FP) in Cas9-only controls (FP-Cas9 only), and Cas9 ribonucleoproteins (RNPs) with sgRNA targeting CD55 in nontreated (FP-RNP) or silane-treated inserts (FP-RNP-Silane). (B) Percentage recovery of Jurkat cells immediately after filtroporation. (C) Fraction of human CD34$^+$ hematopoietic stem and progenitor cells (HSPCs) recovered immediately after filtroporation. (D) Recovery of HSPCs at 24 h after treatment and (E) viability of those cells as determined by trypan blue counterstaining including electroporation (EP) controls (EP-Mock were electroporated without cargo or in the presence of CD55-targeting RNPs for EP-RNP). (F) Number of live Jurkat cells showing proliferation over four days in culture as determined by trypan blue counterstaining. (G) Number of live HSPCs over days in culture, including electroporated controls. (****P <0.0001)

Movie S1 (separate file). Water droplets added to surfaces of filters treated with perfluoroperhydrophenanthrene (PFPP) oil only, without silanization, are stationary and absorb into the filter.

Movie S2 (separate file). Water droplets added to surfaces treated with fluorosilane and later lubricated with PFPP oil (creating a slippery liquid-infused porous surface) show smooth movement across the surface and no absorption.
